# Antioxidant Functionalized Nanoparticles: A Combat against Oxidative Stress

**DOI:** 10.3390/nano10071334

**Published:** 2020-07-08

**Authors:** Harsh Kumar, Kanchan Bhardwaj, Eugenie Nepovimova, Kamil Kuča, Daljeet Singh Dhanjal, Sonali Bhardwaj, Shashi Kant Bhatia, Rachna Verma, Dinesh Kumar

**Affiliations:** 1 School of Bioengineering & Food Technology, Shoolini University of Biotechnology and Management Sciences, Solan 173229, Himachal Pradesh, India; microharshs@gmail.com; 2 School of Biological and Environmental Sciences, Shoolini University of Biotechnology and Management Sciences, Solan 173229, Himachal Pradesh, India; kanchankannu1992@gmail.com (K.B.); rachnaverma@shooliniuniversity.com (R.V.); 3 Department of Chemistry, Faculty of Science, University of Hradec Kralove, 50003 Hradec Kralove, Czech Republic; eugenie.nepovimova@uhk.cz; 4 School of Biotechnology and Biosciences, Lovely Professional University, Phagwara 144411, Punjab, India; daljeetdhanjal92@gmail.com (D.S.D.); sonali.bhardwaj1414@gmail.com (S.B.); 5 Biotransformation and Biomaterials Laboratory, Department of Microbial Engineering, Konkuk University, Seoul 05029, Korea; shashikonkukuni@konkuk.ac.kr

**Keywords:** oxidative stress, antioxidants, nanoparticles, biological nano-antioxidants

## Abstract

Numerous abiotic stresses trigger the overproduction of reactive oxygen species (ROS) that are highly toxic and reactive. These ROS are known to cause damage to carbohydrates, DNA, lipids and proteins, and build the oxidative stress and results in the induction of various diseases. To resolve this issue, antioxidants molecules have gained significant attention to scavenge these free radicals and ROS. However, poor absorption ability, difficulty in crossing the cell membranes and degradation of these antioxidants during delivery are the few challenges associated with both natural and synthetic antioxidants that limit their bioavailability. Moreover, the use of nanoparticles as an antioxidant is overlooked, and is limited to a few nanomaterials. To address these issues, antioxidant functionalized nanoparticles derived from various biological origin have emerged as an important alternative, because of properties like biocompatibility, high stability and targeted delivery. Algae, bacteria, fungi, lichens and plants are known as the producers of diverse secondary metabolites and phenolic compounds with extraordinary antioxidant properties. Hence, these compounds could be used in amalgamation with biogenic derived nanoparticles (NPs) for better antioxidant potential. This review intends to increase our knowledge about the antioxidant functionalized nanoparticles and the mechanism by which antioxidants empower nanoparticles to combat oxidative stress.

## 1. Introduction

In the twenty-first century, age-related diseases have become a major health concern worldwide. Ageing is a natural and progressive process which involves the degeneration of the functioning and structure of vital organs and is one of the risk factors responsible for numerous chronic diseases and accounts for the high mortality rate [1,2,3,4]. Among various theories thatunveil and elucidate the ageing process, the free radical theory holds an exceptional rank [5]. This theory states that the ageing occurs due to successive failure of the defense mechanism to resort the damage induced by the reactive oxygen species (ROS), especially in the mitochondria [6].

**Figure 1:**
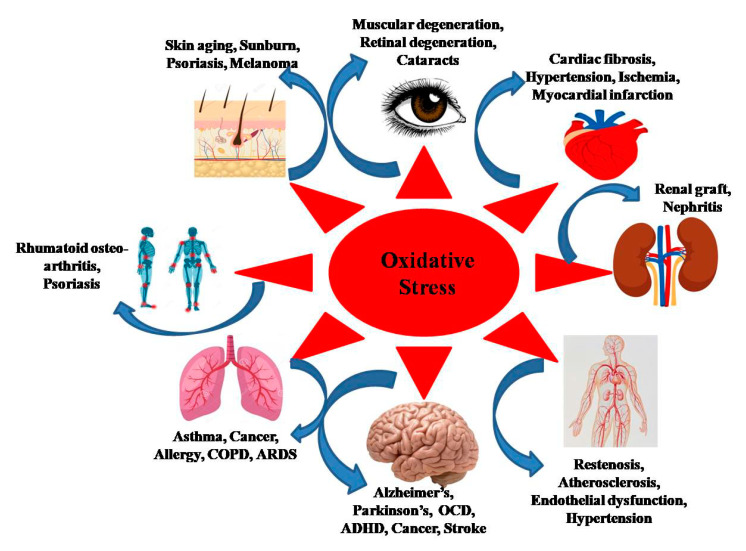
Side effects of oxidative stress on human body. COPD—Chronic obstructive pulmonary disease; ARDS—Acute respiratory distress syndrome; OCD—Obsessive-compulsive disorder; ADHD—Attention-deficit/hyperactivity disorder.

It is well comprehended that oxidative stress plays a significant role in degenerative senescence. ROS have been found to be involved in the pathogenesis of various cellular processes, and is also associated with numerous diseases like cardiovascular, cancer, neurodegenerative and respiratory diseases, as depicted in Figure 1 [7]. The rise in ROS concentration in cells have also been associated with ageing, however, it cannot be considered as the only determining factor responsible for ageing. Moreover, in age-related diseases, the elevated concentration of ROS has been involved in the impairment of mitochondria and cellular oxidative damage [2,8].

The production of ROS generally relies on both enzymatic as well as non-enzymatic reactions. The enzymatic reactions involved in various cellular processes, like phagocytosis, prostaglandin synthesis and respiratory chain system, are known to generate ROS [9,10,11,12,13,14,15,16,17,18,19]. The superoxide radical (O2^•−^) is synthesized during the activity of enzymes, like NADPH oxidase, peroxidase and xanthine oxidase, in various cellular processes. It has also been found that various other ROS, like hydrogen peroxide (H_2_O_2_), hydroxyl radical (OH^•^), hypochlorous acid (HOCl), peroxynitrite (ONOO^−^), etc., are also formed during enzymatic reaction, and the action of enzymes like xanthine oxidase and amino acid oxidase leads to the formation of H_2_O_2_. Furthermore, OH^•^ is regarded as a highly reactive free radical species formed during the “Fenton reaction” between H_2_O_2_ and O2^•−^, in the presence Cu^+^ or Fe^2+^, which acts as the catalyst [11,12,13,14,15,16,17,18]. On the other hand, the non-enzymatic reactions between organic compounds and oxygen, or when cells are exposed to ionizing radiations and during mitochondrial respiration, have also been found to be involved in ROS formation [14,15,18]. At present, extensive research is being conducted to explore the natural compounds that can control oxidative stress and improve the immune system [20]. The search for novel molecules with antioxidant properties is an effective way to promote healthy ageing and counteract oxidative stress. Hence, this review focuses on highlighting the effectiveness of antioxidants functionalized nanoparticles. The first section of the review discusses synergism between ROS and age-related diseases, antioxidants and sources of antioxidants. The section following that discusses the role of nano-antioxidants; antioxidant functionalized nanoparticles and challenges associate with them.

## 2. Synergism between ROS and Age-Related Diseases

The overproductions of ROS have been found to be associated with numerous chronic diseases like cancer, cardiovascular, neurodegenerative and respiratory ailments. The synergism between ROS and chronic diseases is discussed in the following sections.

### 2.1. Cancer

Cancer, a fatal disease involves the malignant growth of tumors because of chromosomal alteration and lead to unregulated growth of the cells [21]. This deadly disease has a complex relationship with ROS and is involved at three different levels of cancer development, i.e., initiation, progression and promotion [22]. During the initiation stage, the ROS causes a mutation in DNA, which keeps on accumulating when the affected tissue does not get repaired [23]. The overproduction of ROS triggers the mutation in an oncogene, which potentially contributes to the initiation of cancer [24].

The cancer cells favor the excessive production of ROS in comparison to healthy cells, because of the alteration in the metabolic processes [25]. ROS-induced oxidative stresses in tumor triggers the cell signaling pathways and build resistance in tumor cells and elevate the supply of blood to tumor cells and promote their metastasis [26]. The elevated level of ROS plays a significant role in expanding tumor cells by altering the genes associated with apoptosis, cell proliferation and transcription factors [27]. Furthermore, ROS also downregulate the pro-apoptotic proteins by interfering with the Akt/PI3K and ERK cell signaling pathway, and upregulate the anti-apoptotic genes [28,29]. During the cancer progression stage, ROS interferes with cellular processes and upregulates the production of metalloproteinases by obstructing the angiogenesis process and, by anti-proteases, results in the metastasis of cancer cells [23,25,30].

### 2.2. Cardiovascular Disease

Cardiovascular disease, another fatal ailment, has a strong association with ROS during the development stage [31]. The overproduction of ROS in vascular cells during the reactions involving enzymes like NADPH oxidase, nitric oxide synthases causes the modification in low-density lipoproteins (LDL) [32]. Furthermore, ROS are also found to be involved in cardiac hypertrophy development, myocyte apoptosis and ischemia-reperfusion injury, which ultimately lead to cardiac arrest [33,34,35].

### 2.3. Neurodegenerative Diseases

Neurons are fundamental units of the brain and play a significant role in coordinating the actions and reactions of bodily functions. These neurons are highly vulnerable to ROS, as they weaken the antioxidants defense system, elevate the fatty acid (polyunsaturated) content in the cell membrane and increase the oxygen demand [36]. The significant research conducted in this direction revealed that ROS generation takes place through numerous mechanisms, and play a vital role in developing neurodegenerative diseases like Alzheimer’s, Huntington’s and Parkinson’s disease. These ROS considerably affects the neuron and other cellular processes, the controlling of ROS level may serve as a potential treatment to restrain neurodegenerative disorder and provide relief from its associated symptoms [37].

### 2.4. Respiratory Disorders

Asthma and chronic obstructive pulmonary disease (COPD) are major respiratory disorders, accounting for high mortality worldwide [38]. Exposure to cigarette smoke and air pollutants significantly contributes to the overproduction of ROS in both asthma and COPD patients. The ROS primarily affects and damages the alveolar and connective tissues of the pulmonary system [39]. The overproduction of ROS also triggers the inflammatory cells, which, as a result, shows production of ROS in the pulmonary system. The ROS are predominantly observed during pathophysiology analysis for both asthma and COPD [40]. It is still unclear how increased ROS is a causative factor for these respiratory diseases [41]. Researchers are extensively working in this direction to decipher the role of these ROS in progression of these fatal diseases.

This synergism between ROS and these chronic diseases shows the major challenge associated with oxidative stress induced by ROS, and requires the effective solution to meet the challenges imposed by the overproduction of ROS.

## 3. Antioxidants

An antioxidant can be described as any substance or compound capable of inhibiting the oxidation of suitable substrate even when present in low concentrations [42]. During the late 19th and early 20th century, the exploration of antioxidants resulted in a boom, due to their involvement in various industrial processes like the prevention of corrosion, the polymerization of fuels, fouling in combustion engines and the vulcanization of rubber [43]. The application of antioxidants was limited for the prevention of the oxidation of unsaturated fats, as it resulted in the rancidity of fats [44]. The general procedure to determine the antioxidant potential of any compound involves the assessment of the rate of oxygen consumption when fat is kept in an enclosed container with oxygen. The identification of vitamins A, C and E, which act as antioxidant agents, has revolutionized the field and highlighted the significance of antioxidants in the biochemistry of living beings [45,46].

Another way to understand the antioxidant is that it is a stable molecule, which donates an electron to unwanted free radical species and neutralizes it, and curbs its ability to cause damage. In general, these antioxidants either inhibit or delay the cellular damage because of their scavenging properties [47]. The low molecular weight of these antioxidants allows them to interact with ROS (free radicals) easily and terminate their chain reaction before damaging vital molecules. Glutathione, uric acid and ubiquinol are few antioxidant molecules that are generated by our body during normal metabolic processes [48]. There are various enzymes are present in our body that can scavenge free radicals, and micronutrients like ascorbic acid (vitamin C), β-carotene and α-tocopherol (vitamin E) [49]. The body cannot produce these micronutrients on its own, therefore, these molecules are obtained from the consumed food.

## 4. Sources of Antioxidants

Dietary supplements are key source of antioxidants, which could aid in maintaining good health and prevent the onset of fatal diseases triggered by ROS. Even though some synthetic antioxidants have been developed, their carcinogenic and toxic nature has prompted the exploration for natural antioxidants like vitamins A, C and E [50]. Additionally, population studies have also revealed that the consumption of fruits, tea, vegetables and wine are a reliable source of natural antioxidants, and are effective in regulating the risk of cardiovascular diseases, which has intrigued researchers to exploring their potential as natural antioxidants [51]. Dietary supplements contain antioxidant compounds in the form of phytochemicals i.e., α-tocopherol, β-carotene, vitamin C, vitamin E and various phenolic compounds [52]. Numerous ethnomedicinal plants, fruits, vegetables, mushrooms and other spices have been well-documented as sources of natural antioxidants, which play a significant role in promoting healthy life and treating various fatal diseases [53].

Phenolic compounds obtained from natural sources are considered far better than synthesized chemicals. Microbes are also being explored for synthesizing organic compounds with antioxidant potential. Various fungal strains have been reported to produce compounds like ellagic acid, ferulic acid and gallic acid under solid-state fermentation and submerged fermentation conditions [54]. All these compounds are known to contain 2–4 reactive hydroxyl groups, which impart them the antioxidant potential. In addition, algae and lichens are active producers of different secondary metabolites, including antioxidants (Table 1).

## 5. Nano-Antioxidants

Antioxidants have been accorded as effective therapeutic and prophylactic agents for various diseases. However, these antioxidants have received very limited success until now, as most of the antioxidants show low permeability, and are poorly soluble in water, demonstrate instability during storage and gastrointestinal degradation, which are some of their limitations [61]. The amalgamation of material sciences with nanotechnology has substantially improved and reduced the free radical synthesis during nanoparticle production in different areas and the nanoparticles synthesized for this purpose are regarded as nano-antioxidants [62,63]. Carbon nanotubes, metal and metal oxide nanoparticles and various types of polymer-loaded antioxidant nanoparticles, have been reported to exhibit antioxidant properties [63]. In the past few decades, various preparation protocols, such as emulsion/solvent evaporation, supercritical fluid technology, solvent displacement method, templating method and nanoprecipitation techniques, have been used for synthesizing nano-oxidants [63]. Some oxide nanoparticles can scavenge the reactive nitrogen and reactive oxygen species (RNS/ROS) and mimic the antioxidant molecule, due to their intrinsic physicochemical properties [64]. In the biomedical field, cerium oxide nanoparticles (CONPs) have gathered special attention for their multi-enzymatic scavenging of ROS and their regenerative abilities [65]. These CONPs have unique properties, like the coexistence in both oxidation states i.e., Ce^3+^ and Ce^4+^, the ability to reversibly switch between both oxidation states and the reduction potential of ~1.52 V [66]. Cerium dioxide as a bulk crystal primarily contains Ce^4+^, but during its reduction to nano-size, substantially enhances the relative amount of Ce^3+^, therefore, leading to higher catalytic activity, in contrast to various biological processes and biological antioxidants [67,68]. Hirst et al. (2013) conducted an in vivo test on mice to assess the antioxidant potential of nanoceria, which were injected intravenously in the subject, and the result of the study revealed that nanoceria significantly decreased the lipoperoxidation after the three weeks, which indicates that CONPs are effective in treating oxidative stress [69]. Caputo et al. (2015) conducted a comparative study to assess the antioxidant potential between CONPs and NAC (N-acetyl-cysteine) and Trolox (soluble analogues of vitamin E) [70]. The results of this study revealed that NAC and Trolox reduced the oxidative 2′-7′-Dichlorofluorescein (DCF) signal triggered by irradiated TiO_2_ nanoparticles, but the antioxidant potential was significantly lower in comparison to CONPs. This result also highlights the stability of CONPs because of their auto-regenerative redox cycle, which allows them to surpass the challenges related to the stability of the antioxidants molecules.

On the other hand, synthetic polymeric NPs have emerged as the promising nano-drug delivery system, as they can encapsulate the therapeutic agent and progressively release the therapeutic compound at the target site. Poly-D, L-lactide (PLA) and poly (lactic-co-glycolic acid) (PLGA) are some examples of synthetic biodegradable polymers that have been approved safe by the European Medicine Agency (EMA) and U.S. Food and Drug Administration (FDA) for administration (Table 2).

Liposomes are also used for delivering the antioxidant agents to the target site. The amphiphilic and biocompatible nature of these liposomes allows them to load both hydrophilic and lipophilic compounds and favor the encapsulation of the water-soluble and water-insoluble antioxidant enzymes [79].

Furthermore, chitosan is the material predominantly used for synthesizing nanoparticles as a sole material or in amalgamation with another [80]. Chitosan shows mucoadhesive properties, which improves the targeted delivery in mucosal surfaces such as intestinal and nasal epithelium [81]. Curcumin encapsulated in nanocarrier and covered and stabilized with chitosan has also been developed and evaluated for free radical scavenging in comparison with free curcumin, and showed the protective effect of chitosan on the antioxidant activity of curcumin [82]. Pu et al. (2014) reported the encapsulation of curcumin antioxidant compounds within the nanocarrier and regulation of release of antioxidant compounds, by changing the pH and oxidative stress of inflamed tissues to increase the overproduction of RNS/ROS synthesized by lipopolysaccharide (LPS)-stimulated macrophage [83].

## 6. Antioxidant Functionalized Nanoparticles

Bacteria, algae, fungi, lichens and plants are known to contain diverse bioactive compounds like terpenoids, alkaloids, polyphenols, phenolic acids etc. These bioactive compounds show potential antioxidant activity, and are known to reduce and stabilize the metallic ions. The diverse types of antioxidant functionalized nanoparticles derived from various biological extracts (Table 3) are discussed in the following sections.

### 6.1. Silver Nanoparticles (AgNPs)

The silver nanoparticles (AgNPs), show a substantial biochemical and catalytic activity, which can be attributed to their significantly large surface area, as compared with other particles with similar chemical structures [134]. The AgNPs synthesis occurs via two steps, at first, Ag^+^ ions are reduced to Ag°, followed by the agglomeration of colloidal silver nanoparticles, to form the oligomeric clusters which are finally stabilized [134]. Biological catalysts (enzymes) are required to reduce the Ag^+^ ions, and the production of AgNPs with characteristic antioxidant properties can be achieved with a variety of plant extracts, as shown in Table 3. Patra et al. (2016) extracted the aqueous portion of watermelon rind (WRA), and used it to synthesize the AgNPs under light-exposed conditions at room temperature [92].

In recent studies, actinobacteria have been identified from various ecosystems that are potential natural producers of AgNPs and the whole-cell biomass and cell free extract of *Streptomyces naganishii* MA7 and *Streptomyces griseorubens* AU2, respectively, were used to synthesize silver nanoparticles with potential antioxidant properties [108,111]. On the other hand, fungal species like *Aspergillus versicolor* ENT7, *Cladosporium cladosporioides* and *Pestalotiopsis microspore* have also been used for the production of antioxidant functionalized AgNPs [117,119,120]. Various researchers have also reported *Ganoderma lucidium* as a potential source of antioxidant functionalized AgNPs [126,128,129]. Venkatesan et al. (2016) prepared the extracts of *Ecklonia cava,* a marine alga known to be a reservoir of phenolic compounds that can act as capping and reducing agents, and used it for the synthesis of silver nanoparticles [59]. Several lichens have also been identified with potential antioxidant properties, and extracts of *Parmeliopsis ambigua*, *Punctelia subrudecta, Evernia mesomorpha,* and *Xanthoparmelia plitti* were used to synthesize AgNPs [132].

The AgNPs synthesized using the leaves extract of *Clerodendrum phlomidis,* are found to have ferric reducing power of 1.63 AU, which is higher than the leaves extract used alone [87]. Whereas, on comparing the IC_50_ value of the extract (i.e., 1920 μg/mL), the AgNPs synthesized using the leaves extract of *Clerodendrum phlomidis* showed higher scavenging activity as confirmed by IC_50_ value (i.e.,55.86 μg/mL). Furthermore, the DPPH radical scavenging activity of AgNPs was found to be dose-dependent, showing the maximum inhibition (85.74%) greater than that of the extract alone. The AgNPs IC_50_ value (9.12 μg/mL) was found to be significantly lower, as compared to the extract (IC_50_ value 388.4 μg/mL) and standard ferulic acid (182.8 μg/mL), which confirmed the AgNPs with high antioxidant potential. In 2019, Das and his colleagues reported that AgNPs synthesized using *Morus alba* leaves extract increased the DPPH scavenging activity to 47.81% against the ~56% of the activity shown by standard ascorbic acid at the same concentration. However, plant extract mediated AgNPs showed 95.08% ABTS^+^ scavenging activity, which is the equivalent to that which was shown by the BTH standard i.e., 95.51% at 100μg/mL, therefore indicating its strong scavenging potential. On the other hand, for nitric oxide scavenging activity, AgNPs displayed 64.04% scavenging, as compared to 45.72% and 88.62% in plant extract and gallic acid standard, respectively, at 100 μg/mL. Similarly, AgNPs at 100μg/mL concentration have shown a significant superoxide scavenging activity of 81.92%, as compared to the 85.35% present in the tocopherol standard [90].

### 6.2. Gold Nanoparticles (AuNPs)

Gold nanoparticles (AuNPs) have attained significant attention, owing to their physical characteristics (size and shape), optical properties and biocompatibility [134]. Gold nanoparticles of varied sizes and diverse morphologies have been employed in the medical sector for various purposes, such as the detection of tumors, and as a carrier for drugs etc. (e.g., Paclitaxel) [134]. Antioxidant AuNPs are mostly derived from extracts of plant parts like leaves and fruits, as shown in Table 3. Markus et al. (2016) reported the identification of *Lactobacillus kimchicus* DCY51^T^, a novel probiotic from Korean kimchi, using an intracellular membrane-bound approach, and synthesized antioxidant functionalized AuNPs from these bacteria [107]. It was observed that the AuNPs formed a capping layer which comprised of several amino acids, while the presence of surface-bound proteins rendered them harmless to cancerous cell lines i.e., human colorectal adenocarcinoma (HT29) and murine macrophage (RAW264.7). As compared with gold salts, biological produced gold nanoparticles were found to be better scavengers of free radicals, especially DPPH. A food inhabiting *Enterococcus* species was identified and used for the production of gold nanoparticles, and was further confirmed through techniques like Fourier transform infrared spectroscopy (FT-IR), UV-vis absorption spectroscopy and transmission electron microscopy (TEM) [109].

Veeraapandian et al. (2012) utilized extracellular proteins of *Escherichia coli* to synthesize gold nanoparticles of anisotropic nature [110]. The size and shape of AuNPs are greatly influenced by the quantity of protein, as extracellular proteins significantly increases the shelf life of nanoparticles by acting as a capping agent, and thereby conferring them with stability. Manjunath et al. (2017) in their study, used a fungal endophyte, *Cladosporium cladosporioides*, obtained from *Sargassum wightii*, seaweed, to synthesize gold nanoparticles [118]. The process of reduction of gold metal salts into gold nanoparticles involved the use of phenolic compounds, and was found to be mediated through the activity of NADPH-dependent reductase enzyme. Lee et al. (2015) used the extracts of a mushroom species, *Inonotus obliquus*, to develop AuNPs without application of deleterious agents [127]. In another study, AuNPs were synthesized from the extracts of *Gracilaria corticate,* a red alga inhabiting in the marine environment and using it as a reducing agent [130]. Sharma et al. (2014) used the desiccated biomass of *Lemanea fluviatilis* to reduce and stabilize the AuNPs [131]. Debnath et al. (2016) developed the gold nanoparticles without the use of extraneous stabilizing and reducing agents by using desiccated biomass of lichens inhabiting in the alpine region of Eastern Himalayas (India) at high altitudes [133]. The gold nanoparticles obtained from lichen *Acroscyphus* sp. comprised of prismatic and multiply twinned quasi-spherical shapes, while those synthesized from *Sticta* sp. were simply multiply twinned and, both of these exhibited antioxidant activity.

### 6.3. Copper Oxide Nanoparticles (Cu_2_ONPs)

In recent years, copper (Cu) has intrigued researchers for its application in nanoparticle synthesis, due to its readily available nature, and characteristics like catalytic, electrical, optical and mechanical properties [135,136,137]. Copper oxide, being a vital inorganic material, is predominantly used in modern technologies, especially in the field of catalysis, ceramics and superconductor applications. Moreover, it can also be used as electrode active materials for the degradation of nitrous oxide with ammonia, and oxidizing carbon monoxide, hydrocarbon and phenol in the formation of supercritical water [138]. The phytogenic synthesis of Cu_2_ONPs possessing antioxidant ability is compiled in Table 3. In 2019, Rajeshkumar and his colleagues also reported a similar radical scavenging property of copper nanoparticles synthesized using leaf extract of *Cissus arnotiana*, when compared with the standard ascorbic acid [102].

### 6.4. Iron Nanoparticles (INPs)

Iron is another imperative material used for synthesizing nanoparticles, because of unique physiochemical properties like high catalytic activity, low toxicity, high magnetism and microwave absorption ability [139,140,141]. The iron nanoparticles (INPs) are categorized into three groups i.e., (a) iron oxide nanoparticles (IONs), (b) iron oxide hydroxide (FeOOH) nanoparticles, and (c) zero-valent iron (ZVI) nanoparticles [142,143,144,145]. These nanoparticles are used for varied applications like bio-separation, bioprocess intensification, drug delivery, environmental remediation, ferrofluids, food preservation, gene therapy, hyperthermia, magnetic targeting, negative MRI contrast enhancement, pigments, stem cell sorting and manipulation, thermal-ablation and lithium-ion batteries [146]. Table 3 shows the plant mediated INPs with antioxidant potential. In 2015, Muthukumar and Manickam observed the high antioxidant potential of *Amaranthus spinosus* leaf extract mediated INPs, because of the presence of amaranthine and phenolic compounds in it, which act as the capping agent [104].

### 6.5. Zinc Oxide (ZnONPs), Selenium (SeNPs) and Nickel Oxide Nanoparticles (NiONPs)

Zinc oxide is a metal oxide generally used in n-type of semiconductors. In recent decades, ZnONPs have drawn significant attention, owing to their widespread application in various fields like electronics, optical and the biomedical sector [147]. The process of production of zinc oxide nanoparticles is cost-effective, safe and easy, and ZnO has been given the status of generally recognized as safe (GRAS) by the US Food and Drug Administration [148,149]. ZnONPs are primarily known for their exceptional semiconducting properties, which can be attributed to the wide band gap of 3.37 eV, high catalytic activity, UV filtering properties and large exciton binding energy of 60 meV, and also have good optical, wound healing and anti-inflammatory properties [147]. ZnONPs have been comprehensively used in the cosmetic industry, in products like sunscreen lotions, for their intrinsic UV filtering properties [150]. They also have widespread applications in the biomedical sector, such as in drug delivery, and also exhibit antidiabetic, antibacterial anticancer and antifungal properties [147]. Chandra et al. (2019) used the leaf extract of *Berberis aristata,* a plant of medicinal importance, to synthesize zinc oxide nanoparticles [95]. Other studies have employed the yeast species, *Pichia kudriavzevii* for the extracellular biosynthesis of ZnONPs and the development of ZnONPs by using the fungal strain, *Aspergillus niger* [116,122].

In addition to these materials, selenium nanoparticles (SeNPs) are also attracting researchers, owing to their enhanced properties like semi-conduction, photoelectrical, photoconduction, catalytic etc., and their potential in optical and electronic instruments. They are known to have lesser toxic effects than selenium (Se) compounds, and are used in the medical field, as they show high therapeutic and anticancer properties [151]. Ramya et al. (2015) used an actinomycetes species, *Streptomyces minutiscleroticus* M10A62, obtained from magnesite mine, to develop SeNPs [98]. In 2012, Torres and his colleagues used *Pantoea agglomerans* isolated from the Camarones River to synthesize Se nanoparticles [113]. In 2013, Li and team members fabricated 6-hydroxy-2,5,7,8-tetramethylchroman-2-carboxylic acid (Trolox) coated surface-functionalized nanoparticles of Selenium (Se@Trolox) with antioxidant potential [152]. Moreover, Se@Trolox was found to block the activation of the AKT and MAPK signaling pathway, cisplatin-induced reactive oxygen species (ROS) accumulation, and DNA damage-mediated p53 phosphorylation in HK-2 cells [152].

Recently, nickel oxide has been found to perform different functions in various fields like biomedicine, electronics and magnetism, owing to its properties like anti-bacterial, anti-inflammatory, eco-friendliness, easy usage and high reactiveness [153]. Being highly reactive, it is readily used for catalyzing various organic reactions, like the *α*-alkylation of methyl ketone, the chemo-selective oxidative coupling of thiols, the hydrogenation of olefins, the synthesis of stilbenes from alcohol through Wittig-type olefination and the reduction of ketones and aldehydes [154,155,156,157,158]. Moreover, it is also found to catalyze inorganic reactions, such as the decomposition of ammonia [159]. Recently, it has been used for developing carbon nanotubes (CNTs) [160]. The plant derived nickel oxide nanoparticles showing antioxidant potential are shown in Table 3.

## 7. Challenges

Apart from the widespread applications of metallic nanoparticles, owing to their size, chemical composition, shape, stability, functionalization, surface coating and purity, they also possess potential toxic effects [161]. Nanoparticles show characteristically distinct cellular uptake mechanisms, and also exhibit the ability to catalyze the synthesis of ROS, thus, leading to ROS associated toxic effects [162]. The size of the NPs is also known to affect the uptake of NPs and their distribution within the cell significantly, and it has been found that in NPs with the same dosage but having distinct sizes; the small-sized NPs are readily internalized within the cell than the large-sized NPs [163]. Moreover, it has been observed that the small-sized NPs possess relatively high reactivity, as they have a large surface area [164]. The size, surface charge and the material type of NPs determine the aggregating efficiency of MNPs, which further influences the internalization of NPs in the cell, and ultimately affects the NPs associated toxicity [165]. A lot of research is being carried out at a global scale to evaluate the NP’s associated toxic effects. Cho et al. (2009) found that polyethyleneglycol (PEG)-coated AuNPs triggers acute inflammation, as well as apoptosis, in the liver after intravenous injection, and also leads to the aggregation of the AuNPs in the cytoplasmic vesicles, liver and spleen [166]. There are numerous transition metals like copper (Cu), chromium (Cr), iron (Fe), silicon (Si) and vanadium (V), which are also involved in the generation of ROS via the Fenton reaction and the Haber-Weiss mechanism [167]. Apart from this, ZnONPs have also been reported to increase the cytotoxicity, due to the formation of ROS, which causes oxidative damage, and evoke the release of inflammatory mediators, thus, resulting in the senescence of phagocytic RAW264.7 cells and the transformation of BEAS-2B (human bronchial epithelial) cells [168]. These are some of the challenges associated with the use of metallic and metalloid NPs, and continuous efforts are being made to eliminate and overcome these challenges.

## 8. Conclusions

A sedentary lifestyle and the consumption of high carbohydrate, proteins and fat have drastically changed the lives of humans, resulting in the production of ROS, which subsequently leads to oxidative stress. Moreover, these oxidative stresses are known to be linked with various diseases, and many attempts have been made to subside these with different medications. Although conventional therapies using antioxidants have been used in the past, they were mostly found ineffective in treating various diseases because of their incompetence in passing through the blood–brain barrier. To overcome these challenges, several antioxidant functionalized nanoparticles have been derived from biological sources, and evaluated by using scavenging assays under invitro conditions. Antioxidant properties of these nanocarriers make it a suitable candidate for targeted delivery, and can open new opportunities for combating oxidative stress in in vivo conditions. Furthermore, researchers are making continuous effort to slow down the toxic effects associated with the metallic and metalloid nanoparticles.

## Figures and Tables

**Table 1:** Antioxidants from different biological sources.

Source	Antioxidants	Ref.
Bacteria	Thiazostatins A, 5-(2,4-Dimethylbenzyl) Pyrrolidin-2-One, Phenazoviridin, Benthophoenin, Benthocyanins A, B and C, Benzastatins C, Benzastatins A, (Z)-1-((1-Hydroxypenta-2,4-Dien-1-Yl)Oxy)Anthracene-9,10-Dione	[53]
Plants	Gallic acid, Protocatechuic acid, p-Coumaric acid, Caffeic acid, Rosmarinic acid, Carnosol, Carnosic acid, Rosmanol, Rutin, Epicatechin gallate, Epigallocatechin gallate, Epicatechin, Quercetin (flavanol), Eugenol, Carvacrol, Safrole, Thymol, Myristicin, Menthol, 1,8-Cineol, α-Terpineol, p-Cymene, Cinnamaldehyde, Piperine, Flavone, Flavonol, Chalcone, Flavanone, Anthocyanin, Anthocyanidin-3,5-glucoside, Alpha tocopherol, Gamma tocopherol, Ascorbic acid, Ascorbyl palmitate, Propyl gallate, Resveratrol	[55]
Fungi	Isopestacin, Pestacin, Atrovenetin, 2-Acetonyl-2,4,9,-Trihydroxy-6-Methoxy-7-Methyl-1HPhenalene-1,3(2H)-Dione, Graphislactone, 4,6-dihydroxy-5-methoxy-7-methyl-1,3-dihydroisobenzofuran, 4,5,6-trihydroxy-7-methyl-1,3-dihydroisobenzofuran, 4,6-dihydroxy-5-methoxy-7-methylphthalide, Kojic acid, Phomapyrone C, Versicolone A, Terremutin, Terreic Acid, Neoechinulin A, Candidusin B, 3″-Dihydroxyterphenyllin and 3-Hydroxyterphenyllin, p-Hydroxybenzoic acid, Protocatechuic acid, Gallic acid, Gentisic acid, Vanillic acid, 5-Sulfosalicylic acid, Syringic acid¸ Veratric acid, Vanillin, Cinnamic acid, p-Coumaric acid, o-Coumaric acid, Caffeic acid, Ferulic acid, 3-O-Caffeoylquinic acid, 4-O-Caffeoylquinic acid, 5-O-Caffeoylquinic acid, Quercetin, Rutin, Kaempferol, Myricetin, Chrysin, Catechin, Hesperetin, Naringenin, Naringin, Formometin, Biochanin, Pyrogallol, Resveratrol, Ellagic acid, Tannic acid, Sinapic acid, Flavonols, Flavones, Isoflavones, Flavanones, Anthocyanidins, Flavanols, Vitamin C, Vitamin E, Homogentisic acid	[53,56,57]
Algae	β-carotene, Lutein, Bromophenol, Carrageenan, Fucophlorethols, Fucoxanthin, Galactan sulphate, Phlorotannins, Phycoerythrin, Porphyran, Shinorine, Catechin, Epicatechin, Gallate, Alginic acid, Laminaran, Vitamin A, Phloroglucinol, Eckol, Fucodiphlorethol G, Phlorofucofuroeckol A, 7-phloroeckol, Dieckol, 6,6′-bieckol, Triphlorethol-A, 2,7′-phloroglucinol-6,6′-bieckol	[58,59]
Lichens	1-Chloropannarin, 2-O-Methylsekikaic acid, Atranol, Atranorin, Barbatic acid, Boninic acid, Chloroatranol, Chloroatranorin, Chlorohematommic acid, Cryptostictinolide, Divaricatic acid, Ergosterol peroxide, Ethyl chlorohematommate, Evernic acid, Fumarprotocetraric acid, Gyrophoric acid, Hematommic acid, Lecanoric acid, Lecanoric acid, Methyl orsellinate, Orcinol, Physodic acid, Protrocetraric acid, Sekikaic acid, Umbilicaric acid	[60]

**Table 2:** In vivo and in vitro delivery of antioxidants by different nanoparticles.

Nanoparticles	Delivered Antioxidant/Enzymes	Method of Preparation	Characterization	Size	Test System	Biological Effects	Ref.
Poly(lactide-co glycolide) (PLGA) and Polycaprolactone (PCL)	Ellagic acid	Emulsion -diffusion-evaporation	DLS, Zeta potential	ND	Overnight fasted male Sprague Dawley (SD) rats	Prevent cyclosporine A (CyA)-induced nephrotoxicity	[71]
Polybutylcyanoacrylate, Liposomes, Poly(Lactide-co-Glycolide)	Superoxide dismutase (SOD)	Emulsion solvent evaporation	DLS, Zeta potential	ND	C57BL/6 mice	Nanoparticles displayed protection against reperfusion injury and ischemia when applied after injury reduced in infarct volume with a 50% to 60%, lowered inflammatory markers, and improved in mice behavior	[72]
Iron oxide (magnetite)	Catalase and SOD	Nanoprecipitation	Zeta potential, TEM	303 ± 38 nm (Catalase loaded), 350 ± 10 nm (SOD loaded)	Bovine aortic endothelial cells (BAEC), Primary human umbilical vein endothelial cells (HUVEC)	Cultured endothelial cells rapidly take magnetically responsive nanoparticles (MNP) under magnetic guidance catalase-loaded providing increased resistance to oxidative stress (62 ± 12% cells rescued from hydrogen peroxide induced cell death vs. 10 ± 4% under non-magnetic conditions)	[73]
Poly(lactide-co glycolide) (PLGA)	SOD	Emulsion solvent evaporation	TEM, DLS, Zeta potential	81 ± 4 nm	Male Sprague-Dawley rats	NPs encapsulated by superoxide dismutase helps in reduction of cerebral injury and promote neurological recovery in a rat cerebral ischemia-reperfusion model	[74]
Bovine serum albumin (BSA)-dextran	Curcumin	Self-assembly	TEM, DLS, Zeta potential	115 nm	Caco-2 cells	At 5 μg/mL curcumin in BSA-dextran nanoparticle the CAA (cellular antioxidant activity) value was 65.35, significantly more that of free curcumin (48.61) at the same concentration, showing that nanoencapsulation increased the uptake of curucmin (*p* < 0.05). Curucmin-loaded BSA-dextran nanoparticle EC_50_ values was 3.27 μg/mL, indicating the CAA of curcumin was enhanced by nanoparticle-based delivery systems	[75]
Mesoporous silica	Caffeic acid, Rutin	ND	TEM, Zeta potential	200 nm	Caco-2 and the epidermal HaCaT cell lines	After 24 h incubation of cells with grafted nanoparticles the best results were given by Rutin in terms of antioxidant capacities preservation during coupling procedures, decrease of ROS level and cellular toxicity alleviation. Rutin protective effects were found more apparated in HaCaT than in Caco-2 cells, revealing much cellular specificity towards defense against oxidative stress; MSN-RUT has ability to stimulate a strong Nrf2 protective response in HaCaT cells, accompanied by a comparable induction of HO-1 mRNA. These responses level in Caco-2 cells was again less important.	[76]
Chitosan/alginate	Quercetin	Gelation	Zeta potential	ND	Human hepatocellular carcinoma HepG2 cells and Male Wistar rats (paracetamol-induced liver injury)	Pretreatment of HepG2 cells with (10 µg/mL) encapsulated quercetin significantly reduction in cell viability in H_2_O_2_-induced oxidative stress (0.1 mM H_2_O_2_), thus showing an efficacious in vitro protection; oral pretreatment with encapsulated quercetin (0.18 mg/kg b.w., 7 days) significantly reduced the increased serum transaminases ALT and AST levels, reduced the lipid peroxidation and restored the gluthation (a marker of cell antioxidant defence system) levels	[77]
Stearic acid- and stearyl ferulate-based solid lipid	Trans-ferulic acid	Microemulsion	DLS, TEM	505 ± 8.2 (SLN-FA), 600 ± 3.4 nm (SLN-SF-FA)	Male rats	Both SLN-SF-FA and SLN-FA dose-dependently reduced lipid peroxidation induced by the three oxidants (NADPH/ADP-Fe^3+^, AAPH and SIN-1). SLN-SF-FA showed high efficiency (EC_50_) and potency (maximal activity) against NADPH/ADPFe^3+^ and AAPH-induced lipid peroxidation	[78]

TEM—transmission electron microscopy; DLS—dynamic light scattering; ND—not defined.

**Table 3:** Antioxidant potential of functionalized nanoparticles.

Antioxidant Source	Types of Nanoparticles	Biological Extract	Temperature	Reaction Time	Characterizations	Morphology	Size	Stability	Antioxidant Activity	Ref.
Plant	Silver	*Lantana camara* L. leaves extract (terpenes rich)	RT	24 h	UV-Vis, XRD, Zeta potential; SEM	Sphere	425 nm	Nd	A10 μL of AgNP (2 mg/mL), spot intensity was found good and comparable with ascorbic acid	[84]
	Silver	*Taraxacum officinale* leaf extract	RT	15 min	UV-Vis, XRD, FTIR, HRTEM	Sphere	15 nm	4 months	The efficiency of AgNPs were found against ABTS radicals, displayed an IC_50_ value of 45.6 μg/mL; scavenging potential of Nitric Oxide is 72.1% at 100 μg/mL concentration with IC_50_ value of 55.2 μg/mL	[85]
	Silver	*Bergenia ciliate crude extract*	RT	3 h	UV-Vis, SEM, FTIR,	Sphere	35 nm	Nd	Results of DPPH activity showed the effective free radical % scavenging potential of *Bergenia ciliate* AgNPs is 59.31%	[86]
	Silver	*Clerodendrum phlomidis* L. leaves extract	RT	10 min	UV-Vis, SEM, TEM, EDAX, FT-IR	Sphere	23–42 nm	Nd	AgNPs exhibited remarkable antioxidant activity than the crude extract using phosphomolybdate assay, ferric reducing power, superoxide radical scavenging activity and DPPH assay	[87]
	Silver	*Hippophae rhamnoides* L. leaves extract	RT	24 h	UV-Vis, TEM, HRTEM, FTIR	Sphere	10–40 nm	1 year	The SBT@AgNPs showed excellent DPPH radical scavenging capacity. The results also revealed that the antioxidant properties of the samples depends on dose as their concentrations (5–25 µg mL^−1^) increase their percentage DPPH radical scavenging abilities also increased	[88]
	Gold	*Hippophae rhamnoides* ssp. *Turkestanica* leaves and berries extract	RT	2 min (LE), 15 min (BE)	UV-Vis, HRTEM, FTIR, XRD	Triangles, hexagon and sphere (BE AuNPs), Sphere (LE AuNPs)	55 nm (BE AuNPs), 27 nm (LE AuNPs)	5 months	Colorimetric DPPH assay at (80 µg/mL) concentration showed, free radical scavenging activity was found maximum in LE AuNPs (81%) and BE (70%) AuNPs. LE AuNPs nanospheres (IC_50_ 49 µg) revealed a little better (14%) antioxidant capacity as compared to BE nanotriangles (IC_50_ 57 µg)	[89]
	Silver	*Morus alba* leaf extract	RT	10 min	UV-Vis, FTIR, SEM, FESEM, EDX, HRTEM, XRD, DLS	Sphere	12–39 nm	Nd	Dose dependent antioxidant activity against free radicals like DPPH, ABTS^+^, superoxide and nitric oxide	[90]
	Gold	*Couroupita guianensis* Aubl. fruit extract	70 °C	60 min	UV-Vis, FTIR, TEM, XRD, DLS, Zeta potential	Cubic	26 nm	45 days	For DPPH assay, CGAuNPs IC_50_ was 37 μg/mL; CGAuNPs were potent in scavenging the hydroxyl radicals with IC_50_ values of 30 and 36 μg/mL respectively; CGAuNPs superoxide scavenging activity increased with increasing concentrations and was observed as 89.8% inhibition rate	[91]
	Silver	*Citrullus lanatus* rind extract	RT	24 h	UV-Vis, SEM, EDX, FTIR, XRD	Sphere	109.97 nm	Nd	AgNPs DPPH free radical scavenging activity at 20–100 μg/mL ranged from 21.65% to 60.97%; AgNPs ABTS radical scavenging activity was 11.25% to 55.26% at a concentration of 20–100 μg/mL; AgNPs Nitric oxide scavenging activity was 9.05% to 54.15% at a concentration of 20–100 μg/mL	[92]
	Silver	*Erythrina suberosa* (Roxb.) leaf extract	RT	Over night	UV-Vis, ATR-FTIR, DLS, TEM,	Sphere	12–115 nm	Nd	AgNPs antioxidant potential was estimated by DPPH radical scavenging assay having IC_50_ 30.04 μg/mL	[93]
	Silver	*Thymus kotschyanus* extract	RT	30 min	UV-Vis, FTIR, XRD, EDS, SEM, AFM, HRTEM	Sphere	50–60 nm	Nd	AgNPs DPPH free radical scavenging activities demonstrate effective inhibition as compared to BHT as the standard antioxidant	[94]
	Zinc Oxide	*Berberis aristata* leaf extract	70 °C	ND	UV-Vis, XRD, FTIR, SEM, EDX, DLS	Needle	90–110 nm	Nd	*B. aristata* leaves extract ZnO nanoparticles showed percent inhibition of 32.06% at the concentration of 1 μg/mL and for 5 μg/mL it was to be 61.63%	[95]
	Gold	*Vitex negundo* leaf extract	RT	ND	UV-Vis, XRD, FTIR, TEM	Sphere	20–70 nm	Nd	Radical scavenging activity of DPPH shown that at a 120 µg/mL concentration, the scavenging activity NPs reached 84.64% and the IC_50_ of the NPs was found to be 62.18 µg; The nitric oxide assay results revealed that the antioxidant property of NPs at a concentration of 120 µg/mL, the NPs scavenging activity reached 69.79% with IC_50_ estimated at 70.45 µg	[96]
	Silver	*Cestrum nocturnum* leaf extract	RT	1 week	XRD, TEM, EDS, SEM, FTIR,	Sphere	20 nm	Nd	AgNPs antioxidant activity for DPPH method was 29.55%	[97]
	Copper Oxide	*Hibicus rosasinensis* leaf extract	RT	48 h	UV-Vis, FTIR, TEM	ND	ND	Nd	Good antioxidant activity from FRAP assay	[98]
	Copper Oxide	*Dioscorea bulbifera* tuber extract	40 °C	5 h	UV-Vis, TEM, EDS, XRD, DLS	Sphere	86–126 nm	Nd	Showed 40.81 ± 1.44%, 79.06 ± 1.02% and 48.39 ± 1.46% scavenging activity against DPPH, nitric oxide and superoxide radicals respectively	[99]
	Copper Oxide	*Adiantum lunulatum* whole plant extract	RT	1 h	UV-Vis, DLS, TEM, EDX, XRD, FTIR	Sphere	6.5 nm	Nd	CAT, APX, and SOD activities have steadily increased according to the increasing concentration of copper nanoparticles treatment to *Lens culinaris*	[100]
	Copper Oxide	*Galeopsidis herba. G. herba* extract	25 °C	24 h	UV-Vis, SEM, FTIR, TEM	Sphere	10 nm	Nd	Showed good scavenging activity against DPPH	[101]
	Copper Oxide	*Cissus arnotiana* leaf extract	RT	4 h	UV-Vis, XRD, SEM, TEM	Sphere	80–90 nm	Nd	The antioxidant property observed was comparatively equal with the standard antioxidant agent ascorbic acid at a maximum concentration of 40 μg/mL DPPH assay	[102]
	Iron	*Amaranthus dubius* leaf extract	60 °C	90 min	UV-Vis, FTIR, XRD, SEM	Sphere	43–220 nm	Nd	Showed high antioxidant activity against DPPH	[103]
	Iron	*Amaranthus spinosus* leaf extract	RT	90 min	UV-Vis, FTIR, TEM, EDX, XRD	Sphere	ND	Nd	Antioxidant efficiency was observed to be 93% against DPPH	[104]
	Iron	*Asphodelus aestivus* Brot. extract	50–60 °C	20 min	UV-Vis, FTIR, TEM, EDX, SEM, XRD	NS	20–25 nm	Nd	Antioxidant activity against DPPH (IC_50_: 3.48 µg/mL) and ABTS (60.52%)	[105]
	Nickel Oxide	*Stevia rebaudiana* Bertoni leaf extract	100 °C	2 h	UV-Vis, XRD, SEM, TEM, FTIR	Sphere	20–50 nm	Nd	Antioxidant efficiency was observed to be 70% against DPPH	[106]
Bacteria	Gold	*Lactobacillus kimchicus* DCY51^T^ biomass	RT	12 h	UV-Vis, FE-TEM, XRD, DLS, FTIR	Sphere	13 nm	NS	Lowest concentration of the biosynthesized AuNps DPPH percentage scavenging ability was 15.85 ± 0.49 and when concentration was increased to 500 μg/mL this scavenging ability was increased to 60 ± 1.82	[107]
	Silver	*Streptomyces griseorubens* AU2 cell free supernatant	RT	48 h	UV-Vis, FTIR, TEM, SEM, XRD	Sphere	5–20 nm	Nd	DPPH free radical scavenging activity of AgNPs showed at various concentrations viz. 9.66, 14.27, 15.59, 23.46 and 54.99%	[108]
	Gold	*Enterococcus* species cell free extract	RT	30 min	UV-Vis, TEM, EDX, FTIR	Sphere	8–50 nm	Nd	AuNPs have ability to scavenge DPPH at all the investigated concentrations (1–40 μg/mL), yielding activities of 33.24–51.47%	[109]
	Gold and Silver	*Escherichia coli* cell protein	RT	ND	UV-Vis, XRD, FTIR, TEM	Triangular, circular, hexagonal (AuNPs), Sphere (AgNPs)	10–100 nm (AuNPs), 10–50 nm (AgNPs)	3 months	EC_75_ (for scavenging 75% effective concentration) of protein capped gold nanoparticles is 916 µg/mL	[110]
	Silver	*Streptomyces naganishii* MA7 biomass	RT	72 h	UV-Vis, FTIR, XRD, EDX, AFM, SEM, TEM, HRTEM	Sphere	5–50 nm	Nd	At a concentration of 1000 μg/mL, AgNPs showed good reducing power comparatively than ascorbic acid (vitamin C)	[111]
	Selenium	*Streptomyces minutiscleroticus* M10A62 biomass	RT	72 h	UV-Vis, XRD, HRTEM, FTIR, EDX	Sphere	10–250 nm	Nd	SeNPs actinobacterially synthesized were found strong free radical scavenging activity compared with standard ascorbic acid was proved by positive DPPH activity. Free radical scavenging activity depends on concentration as increases with increased concentration of SeNPs	[112]
	Selenium	*Pantoea agglomerans* UC-32	RT	24 h	TEM, EDS, SEM	Sphere	100 nm	Nd	High antioxidant activity in human umbilical vein endothelial cells	[113]
	Gold and Silver	*Gordonia amicalis* HS-11 cell free supernatant	100 °C	10 min	UV-Vis, XRD, TEM, FTIR	Grain	5–25 nm	Nd	CFS synthesized AgNPs and AuNPs respectively, showed 88.5 and 87.75% inhibition towards hydroxyl radicals; CFS mediated AuNPs inhibited nitric oxide radicals by 67.5%and with AgNPs the inhibited by 61.5%	[114]
	Silver	*Streptomyces violaceus* MM72 exopolysaccharides	RT	1 h	TEM, EDX, XRD	ND	30 nm	Nd	The DPPH radical scavenging activity shown by SNPs of 89.5% at 50 μg/mL concentration; SNPs exhibited the more total antioxidant activity of 0.730 at 50 μg/mL concentration; H_2_O_2_ scavenging activity of SNPs was evaluated at different concentrations, 50 μg/mL exhibited a higher activity of 72.5% which was significantly higher than that of the standard L-ascorbic acid; SNPs (50 μg/mL) had a nitric oxide scavenging activity of 60.1%; Ferric reducing power of the SNPs was estimated by the reduction of Fe/ferricyanide and the inhibition was observed at 0.390 AU at 50 μg/mL	[115]
Fungi	Zinc Oxide	*Pichia kudriavzevii* cell free extract	RT	36 h	UV-Vis, XRD, TEM, Zeta potential	Hexagonal	10–61 nm	Nd	DPPH radical scavenging activities IC_50_ values were 10 ± 0.52, 5.26 ± 0.42 and 25.46 ± 0.35 µg/mL for ZnO/T1, ZnO/T2 and ZnO/T3 respectively	[116]
	Silver	*Pestalotiopsis microspora* filtrate	RT	24 h	UV-Vis, FTIR, XRD, TEM, DLS	Sphere	2–10 nm	Nd	IC_50_concentrations (the concentration of the sample required to scavenge 50% radicals) of the biosynthesized AgNPs were found to be 76.95 ± 2.96 μg/mL; Biosynthesized AgNPs also exhibited effective scavenging activity against H_2_O_2_ radicals and the maximum scavenging activity of 51.14% ± 1.78% was observed at the highest concentration of 100 μg/mL	[117]
	Gold	*Cladosporium cladosporioides* filtrate	RT	ND	UV-Vis, FESEM, EDX, XRD, FTIR, DLS, AFM	Cubic	100 nm	6 months	DPPH radical scavenging capacity of the AuNPs was found to be dose dependent; AuNPs was subjected to reducing power assay where it showed moderate activity of 1.51 ± 0.03 mg of AAE/g sample	[118]
	Silver	*Cladosporium cladosporioides* filtrate	RT	ND	UV-Vis, FESEM, XRD, FTIR, DLS, AFM	Sphere	100 nm	Nd	AgNPs showed potent antioxidant potential and their radical scavenging ability was increasing with increment in their concentration	[119]
	Silver	*Aspergillus versicolor* ENT7 filtrate	RT	24 h	UV-Vis, TEM, XRD, FTIR	Sphere	3–40 nm	Nd	The radical scavenging activity for the AgNPs at 100 µg/mL was determined as 60.04% which is close to 68.52% obtained for the standard ascorbic acid at the same concentration. IC_50_ value for the AgNPs is found to be 60.64 µg/mL	[120]
	Silver	*Trichoderma atroviride* cell free filtrate	40 °C	ND	UV-Vis, TEM, EDS, FTIR	Variables	15–25 nm	Nd	AgNPs exhibited quite higher DPPH scavenging activity at concentration dependent manner with IC_50_ of 45.6 μg/mL	[121]
	Zinc Oxide	*Aspergillus niger* cell free filtrate	RT	24 h	UV-Vis, FTIR, XRD, DLS, SEM	Rod and cluster	80–130 nm	Nd	Maximum DPPH scavenging of 57.74% was obtained at 100 µg/mL concentration of ZnONPs; ABTS assay scavenging of 73.58% was obtained at 100 µg/mL concentration of ZnONPs	[122]
	Silver	*Penicillium* species extract	RT	10 min	UV-Vis, SEM, XRD	Sphere	18 nm	Nd	FRAP reducing ability potentially reduced by PsAgNPs was 1109.41 where the standard ascorbic acid shows 1648.52 μm	[123]
	Silver	*Inonotus obliquus* extract	RT	80 min	UV-Vis, SEM, EDX, TEM, XRD, FTIR	Sphere	14.2–35.2 nm	8 months	Free radical scavenging activity of the AgNPs on ABTS radicals was found to increase with increase in the concentration, showing maximum inhibition (76.57%) at 1 mM and minimum inhibition (60.98%) at 0.125 mM solution	[124]
	Silver	*Cladosporium* species extract	RT	1 h	UV-Vis, SEM, XRD, FTIR	Sphere	24 nm	Nd	CsAgNPs exhibited potent antioxidant potential and as the concentration increases the radical scavenging ability also increases	[125]
	Silver	*Ganoderma lucidium* and *Agaricus Bisporus* extract	RT and 60 °C	12 h (RT), 5 h (60 °C)	UV-Vis, SEM, XRD, FTIR	Rod	10–80 nm	Nd	EHT synthesized AgNPs shows higher antioxidant activity (75% ± 0.24%) in comparison of standard (70.34% ± 0.03%)	[126]
	Gold	*Inonotus obliquus* extract	RT	30 min	UV-Vis, SEM, EDX, TEM, AFM, XRD, FTIR	Sphere, triangle, hexagonal and rod	23 nm	Nd	ABTS scavenging effect increased with increasing concentration of AuNPs. The ABTS radical scavenging activity showed a maximum and minimum at 1 mM and 0.125 mM, respectively	[127]
	Silver	*Ganoderma lucidium* extract	RT	ND	UV-Vis, TEM, XRD, FTIR, XPS	Sphere	15–22 nm	Nd	DPPH free radicals scavenging activity of AgNPs raised from 32.57% to 54.16% when concentration raised from 50 mg/L to 100 mg/L	[128]
	Silver	*Ganoderma lucidium* extract	60 °C	24 h	UV-Vis, XRD, SEM, FTIR	Sphere	10–30 nm	Nd	Percentage of inhibition by silver nanoparticles showing maximum with 73.49% and minimum with 55.34% at 100 and 10 μg/mL respectively	[129]
Algae	Gold	*Gracilaria corticata* extract	40 °C	4 h	UV-Vis, SEM	ND	45–57 nm	Nd	Synthesized AuNPs revealed a good capacity of DPPH free radical scavenging	[130]
	Gold	*Lemanea fluviatilis* (L.) C.Ag. extract	RT	12 h	UV-Vis, XRD, TEM, HRTEM, DLS, FTIR	Sphere	5–15 nm	Up to 3 months	DPPH scavenging (%) vs. different weights of the sample was found to be 18.10 mg	[131]
	Silver	*Ecklonia cava* extract	RT	72 h	UV-Vis, FTIR, XRD, TEM, DLS	Sphere	43 nm	Nd	250 µg/mL of *Ecklonia cava* extract or biosynthesized AgNPs was mixed with DPPH solution, ca. 50% of scavenging activity was achieved. DPPH radical scavenging activities of *Ecklonia cava* extract and biosynthesized AgNPs were similar at the same concentrations (e.g., 100, 250, and 500 µg/mL)	[59]
Lichens	Silver	*Parmeliopsis ambigua*, *Punctelia subrudecta, Evernia mesomorpha, Xanthoparmelia plitti* mycelia mats	RT	24 h	UV-Vis, SEM, FTIR	Variables	150–250 nm	Nd	SNPs samples total antioxidant capacity are 2.55 ± 0.05, 3.35 ± 0.04, 2.90 ± 0.01, 2.22 ± 0.01 µg AA/g respectively. *Punctelia subrudecta* displayed a higher activity (3.35 ± 0.04 µg AA/g) than the remaining samples; The hydroxyl radical scavenging activity showed that values were 7.75 ± 0.10, 34.48 ± 1.19, 31.97 ± 1.87 and 27.21 ± 1.39 µg/mL for all four samples respectively; Among the tested lichen SNPs, *Punctelia subrudecta* (*Punctelia subrudecta*) gave highest reducing power	[132]
	Gold	*Acroscyphus sphaerophoroides* Lev, *Sticta nylanderiana* extract	RT	12 h	UV-Vis, FTIR, XRD, TEM	Quasi-spherical and prismatic (*Acroscyphus* sp.), Twinned (*Sticta* sp.)	5–35 nm (*Acroscyphus* sp.), 20–50 nm (*Sticta* sp.)	Up to 3 months	1.66 and 4.48 mg sample concentration (SC_50_) were found	[133]

RT–room temperature; Uiv-vis—ultraviolet–visible spectroscopy; TEM—transmission electron microscopy; SEM—scanning electron microscopy; FT-IR—Fourier-transform infrared spectroscopy; XRD—X-ray powder diffraction; EDX—energy dispersive X-ray spectroscopy; DLS—dynamic light scattering; HRTEM—high-resolution transmission electron microcopy; AFM—atomic force microscopy; ND—not defined; NS—not specified; Nd—not determ.
